# IL-7 in SARS-CoV-2 Infection and as a Potential Vaccine Adjuvant

**DOI:** 10.3389/fimmu.2021.737406

**Published:** 2021-09-17

**Authors:** Yonas Bekele, Yongjun Sui, Jay A. Berzofsky

**Affiliations:** Vaccine Branch, Center for Cancer Research, National Cancer Institute, National Institutes of Health (NIH), Bethesda, MD, United States

**Keywords:** IL-7, IL-7R, SARS-CoV-2, COVID-19, adjuvant

## Abstract

IL-7/IL-7R signaling is critical for development, maturation, maintenance and survival of many lymphocytes in the thymus and periphery. IL-7 has been used as immunotherapy in pre-clinical and clinical studies to treat cancer, HIV infection and sepsis. Here, we discuss the critical function of IL-7 in diagnosis, prognosis and treatment of COVID-19 patients. We also summarize a promising role of IL-7 as a vaccine adjuvant. It could potentially enhance the immune responses to vaccines especially against SARS-CoV-2 or other new vaccines.

## Introduction

**Figure 1:**
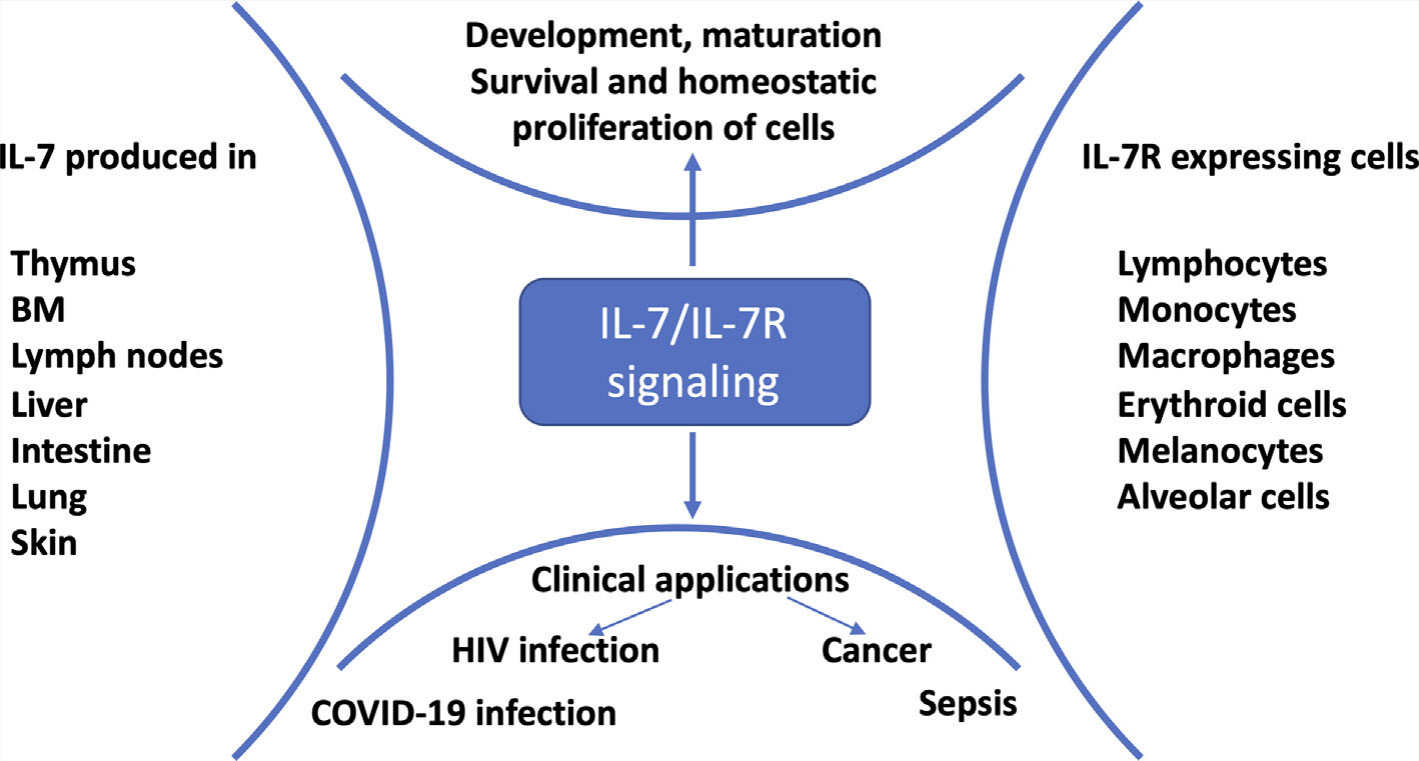
IL-7/IL-7R signaling and clinical applications. IL-7 is mainly produced by stromal cells, epithelial cells, keratinocytes, dendritic cells, follicular dendritic cells and hepatocytes; and consumed by IL-7R expressing cells for development, proliferation, survival and maintenance (1). Preclinical and clinical studies showed that HIV-infected (2), cancer (3), sepsis (4) and COVID-19 (5–7) patients benefited from IL-7 treatment.

Interleukin-7 (IL-7) is a non-redundant and pleiotropic cytokine produced by multiple stromal cells (**Figure 1**). It is critical for development, survival and maintenance of T cells (1, 8, 9). IL-7 binds to the IL-7 receptor (IL-7R), which is a heterodimer structure with an IL-7Rα chain (CD127) and a common gamma chain (CD132) shared with receptors for IL-2, IL-4, IL-9, IL-15, and IL-21 (10). IL-7/IL-7R signaling, prevents memory CD4^+^ T cell apoptosis by increasing levels of anti-apoptotic proteins including Bcl-2, Mcl-1 and Bcl-xL through a JAK/STAT pathway (11). Wallace et al. reported that IL-7 prevents telomere erosion of naïve CD8^+^ T cells cultured for 14 days without losing the replication potential of the cells (12). IL-7 is also important for cell proliferation; it has been shown that IL-7 knockout mice manifested depletion of naïve T cells and a decrease in proliferation, in contrast to IL-4 and IL-15 knockout mice. *In vivo* and *in vitro* T cell proliferation was restored through an exogenous supplement of IL-7 (13). Upon challenge with antigen, naïve T cells differentiate into effector cells, and downregulate the expression of IL-7R; other cytokines take over the regulation of effector T cells (14). When cells return to the resting state after the removal of the challenge antigen, cells upregulate the expression of IL-7R. Thus, IL-7 appears to regulate the survival and homeostasis of memory T cells (14). Pre-and pro-B cells express IL-7R but due to lack of IL-7R in the mature B cells including memory B cells, there is no direct IL-7/IL-7R signaling. However, IL-7 enhanced the expression of CD70 and secretion of B-cell activation factor (BAFF) on T cells which interact with CD27 and BAFF-receptor on B cells, respectively, and thus may induce a better vaccine response (15).

Individuals with Coronavirus disease 19 (COVID-19), which is the disease caused by the Severe Acute Respiratory Syndrome Coronavirus 2 (SARS-CoV-2), presented with a wide-range of symptoms and clinical manifestations. Some patients present with hyper-production of proinflammatory cytokines, also known as cytokine storm, which may contribute to development of acute respiratory distress syndrome (ARDS); this could lead to lung injuries, multiple organ failure and even death. A study conducted in ICU and non-ICU COVID-19 patients in Wuhan, China, reported elevated levels of IL-1β, IL-7, IL-8, IL-9, IL-10, FGF, G-CSF, GM-CSF, IFN-γ, IP-10, MCP-1, MIP-1α, MIP-1β, PDGF, TNF-α and VEGF in patients compared to healthy controls (16). The study also found that plasma IL-2, IL-7, IL-10, G-CSF, IP-10, MCP-1, MIP-1α and TNF-α levels were higher in ICU patients compared to non-ICU patients (16). Similarly Chi et al., investigated the serum levels of cytokines and chemokines in symptomatic, asymptomatic, and convalescent patients, and healthy controls in Jiangsu Province, China (17). Serum levels of IL-7, IL-10 and IP-10 were elevated in COVID-19 asymptomatic cases compared to healthy controls, whereas IL-1RΑ, IL-1β, IL-6 and IP-10 were higher in symptomatic compared to asymptomatic COVID-19 patients (17). This study also reported that IL-6, IL-7, IL-10, IL-18, G-CSF, M-CSF, MCP-1, MCP-3, MIG and MIP-1α levels were associated with disease severity (17). Moreover, serum levels of IL-6, IL-8, IL-10 and CRP were higher in severe COVID-19 patients compared to non-severe patients (18, 19). A study conducted in severe and moderate COVID-19 patients showed that IP-10, MCP-3 and IL-1RA were linked to disease severity and prognostic of outcome of the infection (20). Thus, we summarize here the pathogenic and therapeutic role of IL-7 in COVID-19 patients and highlight the promising role of IL-7 as vaccine adjuvant.

## Elevated IL-7 in SARS-COV-2 Infection

**Figure 2:**
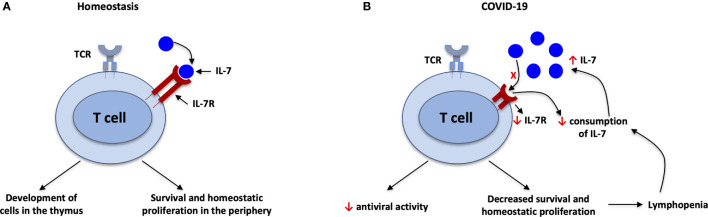
IL-7 during SARS-CoV-2 infection. **(A)** Under homeostatic conditions, there is a balance between IL-7 and IL-7R; IL-7 is important for development of T cells in the thymus and survival of T cells in the periphery. **(B)** Decreased consumption of IL-7 and downgraded expression of IL-7R associated with elevated plasma/serum levels of IL-7 in SARS-CoV-2 infection. IL-7 downregulates its own receptor, IL-7R. Furthermore, antiviral activity and survival of T cells will be decreased due to imbalance of IL-7/IL-7R, leading to lymphopenia. The lymphopenia in turn leads to decreased consumption of IL-7 because T cells are the main consumers of IL-7. This in turn leads to higher circulating levels of IL-7, in a feedback loop.

A low percentage of lymphocytes was measured in all randomly selected deceased COVID-19 patients; however, the percentage of lymphocytes showed a trend of increase in discharged patients. The percentages of lymphocytes were higher than 10% or 20% in patients with severe or moderate symptoms, respectively, compared with lower than 5% in subsequently deceased patients (21). Cytokine storm could be one of the reasons for lymphopenia in COVID-19 patients (21). In chronic HIV-1 infected individuals, elevated levels of IL-7 and lower expression of IL-7Ra were reported as IL-7 downregulates its own receptor (22), and several studies confirmed the link between disease progression and increased plasma/serum levels of IL-7 (**Figure 2**). Elevated plasma/serum IL-7 concentration was also measured during SARS-CoV-2 infection together with other cytokines and chemokines (16, 17, 23, 24). A study conducted in Zurich, Switzerland reported that severe COVID-19 patients exhibited profound loss of naïve T cells and impaired antiviral activity (23). Moreover, serum IL-7 levels were significantly higher in severe COVID-19 patients compared to healthy controls and patient with mild symptoms (23). IL-7 is produced relatively in a constant amount and the level is controlled by consumption, primarily by T cells; elevated circulating levels of IL-7 are associated with depletion of the T cell pool (25, 26) (**Figure 2**). Conceivably, serum IL-7 concentration was also inversely related with the number of T cells, CD4^+^ and CD8^+^ cells; as a feedback response to the lymphopenia, which would further highlight the relation between lymphopenia and elevated IL-7 levels in SARS-CoV-2 infection (23). Impaired circulating innate lymphoid cells (ILCs) were reported in severe COVID-19 patients (27), and these could also be associated with defects in IL-7/IL-7R signaling (28).

In Huang et al., plasma IL-7 levels were higher in ICU patients than in non-ICU patients and healthy adults, supporting the link between disease severity and circulating levels of IL-7 in patients (16). In Lucas et al., COVID-19 patients had lower absolute number and frequency of T cells, CD4^+^ and CD8^+^ cells, and higher IL-7 levels were found regardless of the severity of the disease (24). In a study with COVID-19 patients, elevated serum IL-7 levels were measured in symptomatic and asymptomatic patients compared to healthy controls and convalescent patients (17). This study also measured the serum levels of chemokines and cytokines, and IL-7 levels were elevated in mild, moderate and severe patients compared to controls (17). Moreover, IL-7 concentrations were higher in severe patients compared to the other groups, suggesting use of IL-7 levels as a possible biomarker in measuring the severity of COVID-19 patients (17). Meta-analysis confirmed that male COVID-19 patients have higher risk of hospitalization and even death compared to female ones (29); Chi et al. also confirmed that male patients showed higher IL-7 levels than female patients (17). Altogether, these studies confirm that plasma/serum IL-7 is elevated in COVID-19 patients and may be useful to measure the severity of the disease. In mice, IL-7Ra expression was high in naïve CD8 T cells and was downregulated by cytokine signaling and upon challenge with antigen, and by IL-7 signaling (22, 30). In chronic HIV-infected individuals, IL-7Ra expression was significantly lower than in HAART-treated and healthy controls, suggesting the expansion of CD127 negative T cells in chronic viral infection (31). CyTOF analysis revealed that lower levels of IL-7Ra (CD127) were identified in severe COVID-19 patients compared to mild cases (32); patients who survived from severe SARS-CoV-2 illness showed elevated expression of IL-7Ra (CD127) compared to non-survivors (32). Thus, further studies are warranted to understand the relation between IL-7Ra (CD127) expression and plasma/serum levels of IL-7 in COVID-19 patients. Furthermore, reduced consumption of IL-7 by soluble IL-7Ra (sIL-7Ra) was associated with autoimmune diseases (33); thus a potential link between damage to multiple organs and the levels of plasma/serum sIL-7Ra should be investigated in larger cohort. It is also worth investigating the potential roles of other cytokines in predicting the disease progression and mortality rate of patients with COVID-19.

## Therapeutic Role of IL-7 in Severe COVID-19 Patients

Lymphopenia and lymphocyte exhaustion were reported in severe COVID-19 patients, and linked with outcomes of the infection. One important question is whether the severity of COVID-19 disease can be ameliorated by IL-7 treatment based on lessons learned from studies in HIV-1 infection. A clinical trial in HIV-1-infected adults showed an increase in the T cells upon recombinant human IL-7 (rhIL-7) treatment. The effect was sustained for 45 weeks after treatment interruption and conserved functional properties of T cells (2). Further studies revealed that presence of IL-7 in the culture or *in vivo* induces proliferation of memory T cells (2, 34, 35); this could be due to the decline in the proapoptotic proteins including Bad and Bax (11, 36). In another randomized clinical trial, septic shock and severe lymphopenia patients were benefited from rhIL-7 treatment (4). The absolute lymphocyte counts, and T cell activation and proliferation were increased in rhIL-7 treated patients (4). Moreover, patients who received rhIL-7 did not exhibit further tissue damage, including organ failure (4).

In an examination of case series, critically ill COVID-19 patients who were treated with IL-7 showed increased lymphocyte count without any evidence of hyperinflammation and lung damage (5). In other case studies, increased lymphocyte count and normal IFN score were observed in a severely lymphopenic COVID-19 patient (6) and clinical status was improved in a patient with severe life-threatening conditions after receiving IL-7 (7). Similarly, clinical trials with rhIL-7 therapy for chronically lymphopenic HIV-1 infected individuals showed a dose-dependent increase in CD4 count without increasing immune activation as measured by Ki67 (2, 37). IL-7-treated mice that were infected with lymphocytic choriomeningitis virus (LCMV) showed an increase in the naïve T cell pool compared to PBS-treated mice, suggesting that the treatment enhanced the thymic output and also IL-7-treated mice were able to clear the virus from the reservoirs (38). The mechanism could be the ability of IL-7 to restore the naïve T cell pool through increased survival signals or through more homeostatic proliferation (**Figure 2**); this was also confirmed in clinical trial of rhIL-7 treatment for refractory cancer patients (3). An increase in thymopoiesis induced by IL-7 could also contribute. Patients who received rhIL-7 was able to induce polyclonal T cells and maintained circulating CD4 and CD8 T cells without expansion of T regulatory cells (3). Multicenter clinical trials are recruiting study participants to understand the clinical and immunological benefits of IL-7 treatment in COVID-19 patients (NCT04379076, NCT04407689, NCT04426201, NCT04442178, NCT04927169). IL-7 was safe and tolerable in HIV-1 infected and septic shock patients; and IL-7 also promoted viral clearance in chronic viral infection. Thus, the ongoing clinical trials and new studies should address the safety and efficacy of IL-7, its ability to reduce the viral load, and the survival rate of IL-7 treated COVID-19 patients.

## Adjuvant Effect of IL-7

An IL-7-fused vaccine in animals elicited higher antibody titers than the vaccine alone, with expansion of T follicular helper (Tfh) cells and germinal center (GC) B cells. In contrast, anti-IL-7 treatment showed significant reduction of GC B cells and antibody production (39). Tfh cells are a unique subset of CD4 T cells with B cell lymphoma 6 (Bcl-6) as a master transcription regulatory factor. Tfh cells migrate from the T cell zone to B cell follicles to initiate GC reaction and differentiation of naïve B cells into memory B cells and plasma cells, through upregulation of chemokine (C-XC motif) receptor 5 (CXCR5) and loss of chemokine (C-C motif) receptor 7 (CCR7) (40, 41). Multiple costimulatory molecules are critical for the interaction with follicular B cells in the germinal center (42). Inducible T cell co-stimulator (ICOS), Programmed cell death-1 (PD-1), CD40 ligand, Maf as transcription regulatory factor, CXCL13 and IL-21 cytokine are among the most important of these molecules (41). In mice and monkeys, IL-7-fused trivalent inactivated influenza virus vaccine (TIV) enhanced expansion of Tfh cells and induced higher antigen specific antibodies than vaccine alone (39). IL-7 fusion with vaccine antigen leads to better vaccine response through induction of PD1, ICOS and BCL-6 in the Tfh cells (39). Another study confirmed that administration of IL-7 induced higher levels of transcription factors including *Id3*, *bcl6* and *bach2*, and increased antibody and memory T cells when mice received adeno-associated virus-delivered IL-7 (rAAV-IL-7) with a tuberculosis subunit vaccine (43).

Non-traumatic administration of recombinant glycosylated simian IL-7 (rs-IL-7gly) into the vaginal mucosa triggered over-expression of chemokines and cytokines and enhanced strong antibody responses in macaques (44). The effect was time-dependent since higher expression levels were measured 48 hours after administration than after 24 hours, but the timing may vary with the type of tissue (44). This over-expression of chemokines and cytokines could be due to direct or indirect effects of IL-7, and these chemokines attracted lymphocytes, NK cells, DCs and macrophages to vaginal tissues (44). The study also highlighted the adjuvant role of IL-7 after administrating diphtheria toxoid (DT) vaccine to macaques and measuring the anti-DT antibody. Macaques sprayed in the surface of vaginal mucosa with IL-7 and DT had elevated anti-DT antibody levels compared to macaques without IL-7 pre-treatment (44). The above studies showed the adjuvant role of IL-7 in macaques and mice; in this regard, IL-7 could be a potential adjuvant candidate for adenovirus-based, subunit, inactivated or virus-like particle vaccines in SARS-CoV-2 vaccination studies and clinical trials could consider IL-7 in the future studies. However, a retroviral vaccination study showed that there was no significant change in the vaccine efficacy with co-application of adenoviral vectors encoding Friend Virus with IL-7. Thus, further study is needed to pinpoint the benefits of IL-7 in adenovirus-based vaccines. Additional studies should also address the effect of IL-7 in germinal center (GC) formation, the recruitment of cells including Tfh and GC B cells to GC, upregulation of co-stimulatory molecules and transcription factors. Furthermore, studies should explore the clear mechanism of IL-7 in eliciting the vaccine response in animal models and human clinical trials.

## Conclusions

Clinical trials showed the benefit of IL-7 without severe adverse events and immune restoration in patients with cancer, septic shock and chronic viral infection including HIV. Here, we propose that IL-7 has many potential applications in COVID-19, as a biomarker, as a therapeutic agent, and as a vaccine adjuvant. As mentioned, future studies should highlight the clinical, immunological and virological outcomes of IL-7 therapy in COVID-19 patients. Moreover, prospective clinical trials are needed to validate the biomarker use, to test the therapeutic efficacy at different stages of disease as well as any safety issues and test it further as a component of vaccines. However, its potential role also in cytokine storm or induction of autoimmune disease needs to be kept in mind and excluded during early-stage clinical trials.

## Author Contributions

YB: designed the concept of the work, reviewed the literatures, generated figures, wrote, and edited the paper. YB, YS, and JB: revised the paper. All authors contributed to the article and approved the submitted version.

## Funding

The work was supported by intramural funding under project ZIA-C-004020 and project ZIA-BC-012054 from the Center for Cancer Research, National Cancer Institute, National Institutes of Health, USA.

## Conflict of Interest

The authors declare that the research was conducted in the absence of any commercial or financial relationships that could be construed as a potential conflict of interest.

## Publisher’s Note

All claims expressed in this article are solely those of the authors and do not necessarily represent those of their affiliated organizations, or those of the publisher, the editors and the reviewers. Any product that may be evaluated in this article, or claim that may be made by its manufacturer, is not guaranteed or endorsed by the publisher.
